# FGF23 in Cardiovascular Disease: Innocent Bystander or Active Mediator?

**DOI:** 10.3389/fendo.2018.00351

**Published:** 2018-06-27

**Authors:** Robert Stöhr, Alexander Schuh, Gunnar H. Heine, Vincent Brandenburg

**Affiliations:** ^1^Department of Cardiology, University Hospital of the RWTH Aachen, Aachen, Germany; ^2^Department of Nephrology, University Hospital Homburg-Saar, Homburg, Germany

**Keywords:** FGF23, cardiovascular diseases, heart failure, hypertrophy, left ventricular, myocardial infarction

## Abstract

Fibroblast growth factor−23 (FGF23) is a mainly osteocytic hormone which increases renal phosphate excretion and reduces calcitriol synthesis. These renal actions are mediated via alpha-klotho as the obligate co-receptor. Beyond these canonical “mineral metabolism” actions, FGF23 has been identified as an independent marker for cardiovascular risk in various patient populations. Previous research has linked elevated FGF23 predominantly to left-ventricular dysfunction and consecutive morbidity and mortality. Moreover, some experimental data suggest FGF23 as a direct and causal stimulator for cardiac hypertrophy via specific myocardial FGF23-receptor activation, independent from alpha-klotho. This hypothesis offers fascinating prospects in terms of therapeutic interventions, specifically in patients with chronic kidney disease (CKD) in whom the FGF23 system is strongly stimulated and in whom left-ventricular dysfunction is a major disease burden. However, novel data challenges the previous stand-alone hypothesis about a one-way road which guides unidirectionally skeletal FGF23 toward cardiotoxic effects. In fact, recent data point toward local myocardial production and release of FGF23 in cases where (acute) myocardial damage occurs. The effects of this local production and the physiological meaning are under current examination. Moreover, epidemiologic studies suggest that high FGF-23 may follow, rather than induce, myocardial disease in certain conditions. In summary, while FGF23 is an interesting link between mineral metabolism and cardiac function underlining the meaning of the bone-heart axis, more research is needed before therapeutic interventions may be considered.

## Introduction

Despite relevant progress in diagnosis and therapy, mortality in patients with chronic kidney disease (CKD) and end-stage renal disease (ESRD) remains high, with an overall 65% 5-year mortality (1). Mortality and morbidity associated with CKD are mainly driven by a vast increase in the rates of cardiovascular events. Indeed, the risk of a patient with early CKD to develop cardiovascular disease (CVD) is ~20 times higher than to finally require renal replacement therapy (2). Although traditional cardiovascular risk factors including hyperlipidemia, diabetes, and hypertension are highly prevalent in patients with CKD (3), they cannot alone explain the high cardiovascular disease burden in CKD patients. Several “non-Framingham” or “non-traditional” risk factors have been proposed to contribute to this exploding cardiovascular risk, among which parameters of the so-called CKD—mineral and bone disorder (CKD-MBD) may be of particular importance (4). Many of these parameters link all three entities of the CKD-MBD syndrome, i.e., renal, skeletal and cardiovascular disease. Some of the “non-traditional” cardiovascular risk factors within the spectrum of CKD-MBD such as hyperphosphataemia (5), hypo-and hypercalcaemia (6) as well as secondary hyperparathyroidism (7) have been known for a long time to predict cardiovascular mortality in CKD patients (4). Fibroblast growth factor-23, FGF23 is a rather novel player within the broad spectrum of the CKD-MBD syndrome (8).

## The specific cardiovascular pathology of CKD patients

The cardiovascular disease spectrum of CKD and ESRD patients is specific in various aspects. In addition to accelerated cardiovascular calcification the central characteristic feature of CKD-associated CVD is the development of left-ventricular hypertrophy (LVH). The pathophysiology of CKD-associated LVH is complex and multifactorial and LVH occurs even in the absence of severe and long-standing uncontrolled arterial hypertension or aortic valvular disease in CKD patients (9). Instead, increased vascular stiffness, anemia, hypervolaemia, activation of the renin angiotensin, and of the sympathetic system as well as the toxic effects of uremia-associated circulating factors also contribute to the development of LVH in the setting of CKD (9). Among the latter, FGF23 is a candidate undergoing an intense debate.

## The evolution of FGF23 research over time

An important milestone in our understanding of the bone-heart axis was the discovery of the profound metabolic, specifically mineral effects of FGF23 (10). FGF23 is a 32 kDa bone-derived potent regulator of vitamin D and phosphate metabolism. It was originally identified only some 15 years ago in the osteological community as a phosphaturic hormone in renal phosphate wasting syndromes such as oncogenic osteomalacia (11). Shortly thereafter, it was found that plasma concentration of FGF23 rises dramatically with increasing severity of CKD (12), and moreover, is independently associated with poorer outcome among non-dialysis CKD patients (13, 14) as well as dialysis patients (15). Soon, FGF23 left the nephrology niche and proved to be of particular interest for the cardiology community, since the dismal association between high FGF23 levels and poor prognosis is also detectable in patients selected primarily via cardiac disorders (16–18). Abundant cohort studies followed, all pointing toward the same direction—a strong association between elevated FGF23 levels, cardiovascular morbidity and mortality (19). Of note, FGF-23 turned out to be a stronger predictor of heart failure decompensation rather than of acute atherosclerotic cardiovascular events (14, 20).

The next evolutionary step in our FGF23 understanding was the transfer from association toward causality: Recent experimental data established a causal pathway and linked extra-cardiac FGF-23 directly to the development of cardiovascular pathologies, specifically cardiomyopathy (21). However, as discussed below, even this point of view is again moving forward as evidence grows that the cardiovascular system itself may be able to affect FGF23 levels with potential, yet to be determined, local and systemic effects.

## Physiology of FGF23

**Figure 1:**
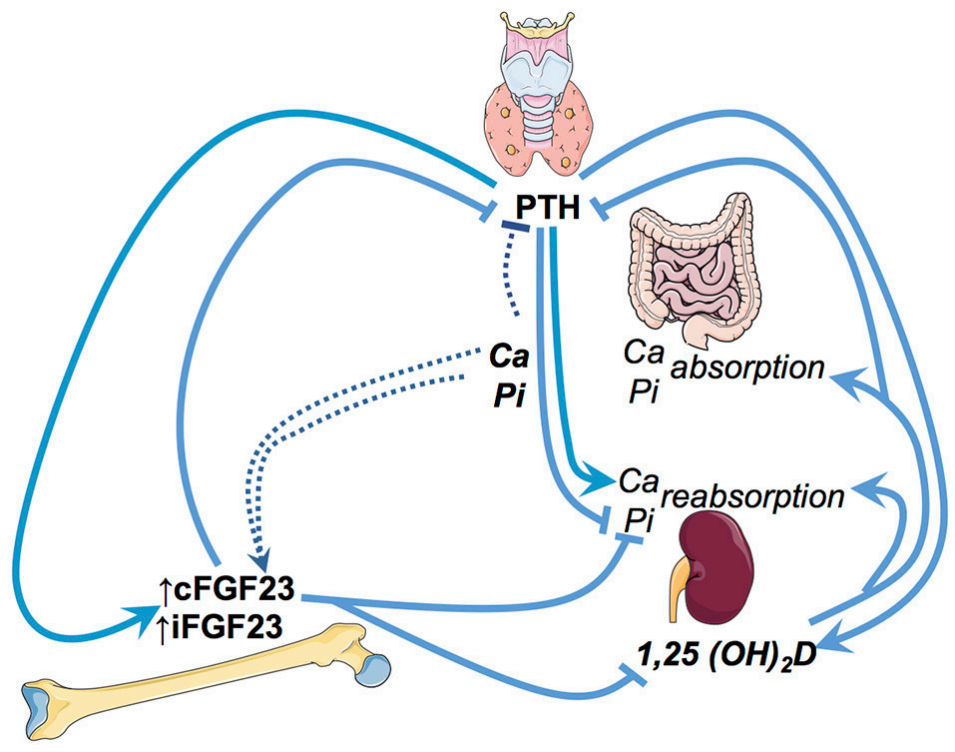
Illustration of the key regulatory pathways of FGF23 in phosphate and vitamin D metabolism. Ca, Calcium; Pi, Phosphate; iFGF23, intact FGF23; cFGF23, c-terminal FGF23; PTH, parathyroid hormone.

Circulating FGF23 is mainly produced by osteocytes and osteoblasts. FGF23 primarily targets the renal tubular and parathyroid cells. These canonical effects of FGF23 (i.e., regulating phosphate, vitamin D metabolism and PTH) depend upon its interaction with FGF23 receptors and its obligate renal co-receptor alpha-klotho (22). In renal tubular cells, upon binding to the FGF-receptor and alpha-klotho—FGF23 (1) stimulates the excretion of phosphate, (2) reduces the activation of calcidiol to calcitriol, and (3) increases the degradation of the latter (23) Hypothetical non-canonical renal effects of FGF23 include the stimulation of distal tubular sodium and calcium absorption (24, 25) as well as suppression of angiotensin converting enzyme 2 transcription in the kidney (26). Furthermore, *in vitro*, FGF23 treatment of bovine parathyroid cells inhibits PTH secretion (27) (summarized in Figure 1).

There is a striking association between decreasing glomerular filtration rate (GFR) and raising FGF23 levels in humans. In fact, the magnitude of FGF23 increase with the decline in kidney function is unique among the various biomarkers of the CKD-MBD syndrome. While a PTH increase by a factor of 20–30 may already represent severe, uncontrolled hyperparathyroidism, FGF23 levels over 1,000-fold higher than in healthy controls have been described in CKD (28).

## FGF23 regulation: phosphate and beyond

The underlying mechanisms of the tremendous rise of FGF23 in renal insufficiency remain partly unclear. Various endogenous, but also external factors contribute to FGF23 regulation such as the phosphorus load and active Vitamin D [1,25 (OH) _2_D3] which appear to be main stimulators of FGF-23 synthesis (29, 30), while other factors including calcium, parathyroid hormone, inflammation, and iron are also involved (31).

Indeed, the exact mechanistical evidence through which decreasing GFR and/or high serum phosphate can increase FGF23 concentrations is still lacking. In fact, it appears an oversimplification to establish a smooth and direct link between phosphate load and/or retention leading to FGF23 increase or vice versa, phosphate restriction, and/or: excretion to FGF23 decrease. Animal and *in vitro* experiments have to date yielded conflicting results. In mice, feeding a high phosphate diet has been shown to increase circulating FGF23 (32), and FGF23 levels correlated positively with levels of circulating phosphate in patients with ESRD (10). However, dietary phosphate modulation has some corresponding effects on circulating FGF23 levels in some studies (33, 34), while other studies found no effect (12). *In vitro*, cultured osteoblast increased FGF23 production in response to 1,25 (OH) _2_D and PTH (35) but not to phosphate (36). In a mouse model of progressive renal failure, Zhang et al. found that severe phosphate restriction (0.2 and 0.02%) did not modulate serum levels of FGF23 (37) despite the expected severe reduction in renal phosphate excretion.

Beyond factors that affect osteoblastic and osteocytic FGF23 production, FGF23 levels and actions undergo modifications via changes in its release (38), cleavage (39), distribution, and allocation of receptors and co-receptors. Cleavage of FGF23 by a furin pro-protein convertase into its N- and C-terminal fragments appears to be the main regulator of its biological effects (40). To date, only intact FGF23 has clearly been shown to have a physiological effect, while the function of the C-terminal fragments remains more controversial (41). Goetz and coworkers have shown that c-terminal FGF23 fragments actually inhibit the FGF23-FGF-R-klotho-interaction (41) and thus antagonize phosphaturic effects of intact FGF23. Hence, FGF23 cleavage is a relevant metabolic step regulating FGF23 activity.

The distribution of FGF-receptors is a major determinant of FGF23 function and is responsible for mediating canonical (renal and phosphaturic) and (e.g., cardiac) effects. FGF-Receptor 1 (FGF-R1) is thought to be the major renal effector of FGF23, mediating primarily phosphaturic and calcitriol-regulatory effects of FGF23 (42). Klotho as obligate co-receptor is of major importance regarding renal mode of action of FGF23 (22).

## Cardiovascular effects of raised FGF23

As already mentioned, over the last years many studies have shown significant and independent associations between increased FGF23 and dismal outcomes in humans. Remarkably, this association is not limited to CKD patients (non-dialysis CKD (13, 14) and ESRD (15, 43)), but also detectable in patients without overt CKD (16–18, 44) in whom no primary significant FGF23 excess is to be expected. While there appears to be a strong and independent association between FGF23 levels and increased mortality as well as heart failure, the association to the development of atherosclerosis is much less pronounced (14, 20) suggesting that occlusive atherosclerotic disease may not be the primary link between increased FGF23 levels and mortality (45). Numerous additional explanations have been proposed as basis for the prognostic impact of FGF23 on (cardiovascular) mortality, which comprise a contribution of raised FGF23 to endothelial dysfunction (46), stimulation of the renin-angiotensin system (47), arterial stiffness (46), vascular calcification (48), inflammation (31), and left-ventricular hypertrophy.

## Specific focus upon FGF23 as the driving force for LVH

Left-ventricular dysfunction has gained specific interest regarding its association with increasing levels of FGF23 now representing an outstanding research field in cardiorenal medicine, since LVH is highly prevalent in CKD and is presumably among the major driving forces for sudden cardiac death (SCD) (49). SCD in turn is among the leading causes of death in CKD patients and the single most prevalent cause for CV death—more prevalent event than classical atherosclerotic cardiovascular events in these patients (50).

Indeed, LVH is a very common finding in patients with CKD and ESRD with up to 74% of patients showing some form of this pathological cardiac remodeling (51–53). The exact prevalence of LVH most likely varies with duration and severity of CKD as well as with the sensitivity of the detection method. Actually, virtually all patients with severe non-dialysis CKD or ESRD will have at least subtle—if not substantial—changes of left ventricular function, geometry, and structure. Evolving and more sophisticated echocardiography and magnetic resonance imaging techniques will make detection of subclinical myocardial changes more sensitive in future. LVH not only predisposes to sudden cardiac death (49), but also increases the risk of developing heart failure: heart failure with preserved ejection fraction (HFPEF) as well as heart failure with reduced ejection fraction (HFREF).

Recent research has attributed a substantial role in LVH pathogenesis to FGF23: As early as 1997 Nehme et al. hinted at it by showing that children with X-linked hypophospataemic rickets (XLH), an X-linked dominant disease with FGF23 overexpression and consecutive hypophosphataemia, to have signs of LVH (54).

In much less selected patients, evidence for an association between FGF23 and LVH was first published by Mirza et al. who showed that in the PIVUS cohort, a Swedish cohort with around 800 patients aged 70 or more, intact FGF23 correlated with the presence of LVH, as determined by echocardiography (55). Noteworthy, this relationship held true, even for FGF23 levels within the normal range. The effects of increased FGF23 were then prospectively analyzed by Faul et al. in a large cohort of 3070 non-dialysis CKD patients. The authors found baseline levels of c-terminal FGF23 to positively correlate with the presence of LVH. Increased FGF23 baseline levels were also associated with an up to 7-fold increased incidence of LVH development in the following 3 years (56). However, there are also studies in hemodialysis patients that have found no association between FGF23 and ventricular mass (57, 58).

In continued research, mainly driven by Faul and coworkers (21, 56, 59–61), several experimental settings were used to determine whether the FGF23 association with LVH was causative or purely associative. The authors concluded that FGF23 is within the causal pathway of CKD-associated LVH development. *In vitro*, the treatment of neonatal rat ventricular myocytes for 48 h with different concentrations of FGF23 induced morphometric hypertrophy similar in extent to treatment with FGF2, a known strong inducer of LVH. These histological changes accompanied a change in the gene expression profile reflecting pathological hypertrophy. The effect was dose dependent, with no further increase seen after 15 ng/ml FGF23 (56). Interestingly, the authors concluded that the cardiac effects of FGF23 were Klotho independent since Klotho was not expressed by cardiac myocytes.

*In-vivo* experiments using intravenous and intramyocardial injection of FGF23 also showed induction of LVH in non-CKD mice (56). Again, Klotho did not appear to be necessary for the cardiac effects of FGF23. Using Klotho-null mice, Faul et al. showed that even in the absence of Klotho, treatment with FGF23 induces LVH to a similar manner as seen in wild type mice. Interestingly, mice heterozygous for Klotho, in which FGF23 raises less pronouncedly than in Klotho null mice, also develop LVH, though to a lesser extent than klotho null mice. While the authors state that this reflects a dose dependent effect of FGF23, it may also reflect the total loss of protective klotho (56).

To further examine the interplay between FGF23 and its cardiac receptors Faul et al. also investigated the effect of FGF23 on FGFR4 null mice and found that abrogation of the FGFR4 prevented the development of LVH *in vitro* as well as *in vivo* while overexpression of FGFR4 induced LVH in non-CKD mice (21). These data raise the hypothesis that the myocardial FGF23 effects are mediated predominantly through the FGFR4 activation of PLC/calcineurin/NFAT pathway. However, it remains unclear if the proposed cardiac effects of FGF23 are all direct effects upon the myocardium or in part reflect systemic FGF23 effects since administration of FGF23 has been suggest to raise blood pressure (24), potentially contributing the induction of LVH (56). Also, XLH-children do not universally exhibit LVH despite chronically raised FGF23 levels (54, 62). Hence, all these findings warrant critical reappraisal and indeed, very recent data point into a different direction.

## FGF23 and myocardial disease: chicken or egg?

The postulated absence of klotho from the myocardium has been the subject of some debate (23), since klotho is essentially required for FGF23 to exert its renal effects (47). Its role in the setting of cardiomyocyte FGF23 toxicity thus remains controversial: While Faul reported a Klotho-independent effect of FGF-23 on cardiomyocytes, other groups suggested that klotho deficiency rather than a FGF23 excess causes cardiac hypertrophy (63): In heterozygous klotho-deficient CKD mice, the development of LVH was not modified by interventions normalizing FGF23 and phosphate levels, but only via exogenous klotho application. In humans, it is currently impossible to clearly separate the effects of CKD-induced klotho deficiency, phosphate load, and FGF23 raise, particularly as no reliable assays for soluble klotho measurements are available so far.

**Figure 2:**
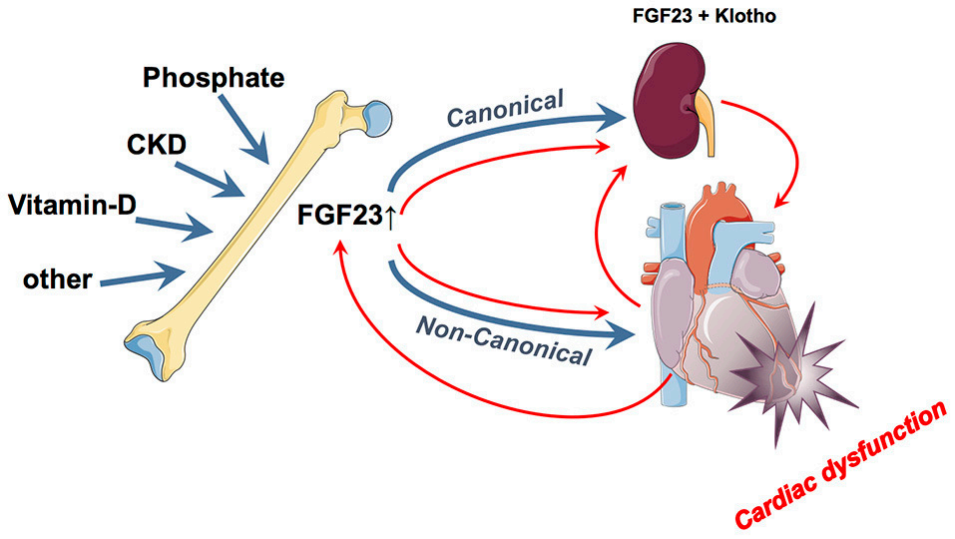
Summary of the currently hypothesized canonical and non-canonical effects of FGF23 on the myocardium and vice-versa. In blue is illustrated the “bone damages heart via FGF23 model” while the red pathways illustrate the “heart damage induces FGF23 alterations model”.

FGF23 has traditionally been believed to be mainly of skeletal origin from which it affects systemically its canonical kidney and parathyroid gland as well as non-canonical targets such as cardiomyocytes (64). While above-mentioned epidemiological data and experimental findings accuse FGF23 (presumably from skeletal origin) as being directly noxious to myocardial cells and being a causative agent for LVH induction and myocardial damage, some data point toward a different or even opposite direction (Figure 2). Two major unsolved issues emerge in this respect: First, does the FGF23, which may finally act upon the myocardium, only originate from the bone or also directly from the myocardium and second, are these actions only deleterious or are there some (dose- and time-dependent) beneficial effects detectable?

Indeed, some beneficial cardiovascular effects have been attributed to FGF23: Remarkably, acute elevations of FGF23 were shown to be positively inotropic: FGF23 induced acute elevations of intracellular calcium of primary cardiomyocytes, an effect that could be abrogated by the calcium channel blocker verapamil. FGF23 treatment of a ventricular muscle strip led to increased contractility, which was blocked by inhibition of FGF receptors (65). Hypothetically, this finding allows speculations about a potential physiological role of FGF23 elevations in situations of acute cardiomyocyte stress. If these effects are (patho) physiologically relevant, it appears counterintuitive that skeletal FGF23 is the sole source for such cardiac actions, because this would require a fast myocardial-skeletal messenger.

In fact, recently Richter el al found cultured cardiomyocytes to express FGF23 (66). In subsequent clinical studies, FGF23 was shown to be present in the explanted hearts of patients with ischaemic or dilated cardiomyopathy undergoing heart transplantation, but not in healthy hearts (67). In another set of experiments, Andhrukova et al. found that, in the setting of experimental myocardial infarction in mice, circulating FGF23 is increased in the circulation with a concomitant reduction of 1,25(OH)_2_D3 (68). Additionally to increased FGF23 production in the bone, myocardial FGF23 was also increased on a protein and mRNA level suggesting that increased circulating FGF23 post-myocardial infarction is at least partly derived from the myocardium itself (68). To add to the complexity of the FGF23-myocardium interaction experiments with the pressure overload model of transverse aortic constriction (TAC) demonstrated that LVH profoundly increased serum levels of intact FGF23, augmented cardiac mRNA and protein expression of FGF23, and increased FGF23 transcription in bone by an aldosterone-driven mechanism (69).

These experimental data are supported by some clinical data fueling the hypothesis that FGF23 release and myocardial damage is not a one-way route from the skeleton to the myocardium: Cross-sectional data derived from a cohort study and *post-hoc* subgroup analyses from a randomized trial on mechanical assist device implantation support the hypothesis that the heart is an active player—not only a recipient—in FGF23 metabolism (70, 71): these patients had much higher FGF-23 than healthy individuals or patients with myocardial infarction not complicated by heart failure. Moreover, among these patients with cardiogenic shock, increased levels of FGF23 on admission correlated with increased mortality at 30 days and 1 year (70). Considering the speed and the magnitude of the FGF-23 rise we speculate that the myocardial damage *per se* induces FGF23 (be it heart-derived and/or bone derived). Hence, this challenged the mono-directional hypothesis that external factors stimulate bone FGF23 release which in turn induces myocardial damage.

Recent data by Anderson et al. underlines how much this topic is currently still up for debate. The authors found that, in a group of patients with acute heart failure, FGF23 is sharply elevated in the circulation (72). In contrary, this does not appear to hold true for chronic cardiac damage as Richter et al. found no upregulation of FGF23 in explanted hearts of patients undergoing heart transplantation for chronic severe heart failure (66).

## Is modification of FGF23 a promising therapeutic option in cardiac disease?

Since many data and researchers suggest that FGF23 excess negatively impacts the cardiovascular system, it appears a potential treatment modality to reduce the circulating amount or to block the FGF23-target organ interaction. However, there are at least two relevant arguments that pharmacological FGF23 blockade is not as straightforward as one could assume. First, as mentioned above, some (early) rise in FGF23 (presumably specifically FGF23 produced and released from the myocardium itself) might have stabilizing effects in the setting of acute myocardial damage. Second, the optimal level and the most appropriate tool of intervention to lower (systemic) FGF23 are unclear.

Clear warning signals against an unreflected (unspecific) blockade emerge from *in vivo* experiments by Shalhoub et al. The authors showed that pharmacological blockade of FGF23 using a pan-blocking antibody failed to prevent the development of LVH in uraemic rats (73). Not only were these results in conflict with the results obtained by Faul et al. (56) but worryingly, FGF23 blockade led to increased aortic calcification and even mortality in the animals (73). The authors attributed this detrimental finding to the raise in serum phosphorus levels which occurred with the blockade of the potent phosphaturic actions of FGF23. Similar results were found when inhibiting FGF23 signaling by a pan-FGFR inhibitor which resulted in increased phosphorus and FGF23 levels leading to multifocal, multiorgan soft tissue mineralization (74). These data clearly remind us that some (systemic) FGF23 actions are beneficial—e.g., the avoidance of phosphate toxicity. Hence, a more specific mode of action such as blocking chronically stimulated FGF23-receptor interactions at the myocardium might hold promise in this respect. Hypothetically, restoration of klotho levels might help to re-direct FGF23 actions toward canonical (renal) signaling pathways and block off-target signaling. Potential effects of FGF23 lowering in humans were shown in the EVOLVE trial in which the use of the PTH-lowering agent Cinacalcet was compared to placebo in patients with terminal renal failure on haemodialysis. Noteworthy, the aim of the intervention was not directly to influence FGF23 synthesis or release but primarily targeted hyperparathyroidism. In this trial, patients receiving cinacalcet showed a significant reduction in mean FGF23 levels (75). Furthermore, patients that responded with a prominent FGF23 decrease of > 30% showed a pronounced reduction in cardiovascular mortality in the 20 week follow-up (75). Against a direct pathophysiological contribution of FGF23 stands that within the placebo group, patients who had a reduction of FGF23 during the study period had no better prognosis than patients who did not have such FGF-23 reduction. Currently, a fully human monoclonal IgG1 antibody, Burosumab, is under investigation for rare diseases with primary FGF23 excess and the clinical picture of phosphate-losing osteomalacia (X-linked hypophosphatemia). In such diseases, where a primary FGF23 excess hits a physiological kidney function, the approach really holds promise and allows to normalize hypophosphatemia. However, a broader application is currently not appealing since chronic effects of FGF23 blockade in conditions with reactive, secondary FGF23 excess are unpredictable.

## Cardiac FGF23 research: the next level

In summary, the jury is still out on the exact role of FGF23 in the development of cardiovascular abnormalities. The FGF23-klotho axis and pathophysiological cardiac effects are a constantly evolving field. Each players' role (klotho, local and systemic FGF23, FGF23-receptors) needs to be exactly determined. Facing the massive threat induced by left-ventricular dysfunction to patients with CKD and ESRD and the potential direct and indirect involvement of low klotho, high FGF23 levels, together with other features of CKD-MBD in this setting an urgent roadmap for further research emerges.

Prior to any therapeutic intervention with the aimtominimize potentially negative FGF23 effects upon cardiac structure and function, research needs to focus on and clarify relevant unsolved issues. Just to name a few, the community needs to prove how cardiac disease induces (rather than follows) FGF-23 secretion, to what degree cardiomyocytes may themselves produce FGF-23 in health and disease, whether such locally produced FGF-23 has a physiological role in (acute) myocardial damage; and whether or not (systemic) FGF23 excess itself directly drives the development of myocardial damage. Only when these questions have been answered, can we try to discuss whether and how to intervene on serum FGF-23 level.

## Author contributions

All authors listed have made a substantial, direct and intellectual contribution to the work, and approved it for publication.

### Conflict of interest statement

The authors declare that the research was conducted in the absence of any commercial or financial relationships that could be construed as a potential conflict of interest.
