# Corrigendum: FGF23 in Cardiovascular Disease: Innocent Bystander or Active Mediator?

**DOI:** 10.3389/fendo.2018.00422

**Published:** 2018-07-18

**Authors:** Robert Stöhr, Alexander Schuh, Gunnar H. Heine, Vincent Brandenburg

**Affiliations:** ^1^Department of Cardiology, University Hospital of the RWTH Aachen, Aachen, Germany; ^2^Department of Nephrology, University Hospital Homburg-Saar, Homburg, Germany

**Keywords:** FGF23, cardiovascular diseases, heart failure, hypertrophy, left ventricular, myocardial infarction

In the original article, there was an error. The last paragraph contained several mistakes carried on from a previous version.

A correction has been made to the section Cardiac FGF23 Research: The Next Level, paragraph 2 and should read:

“Prior to any therapeutic intervention with the aim to minimize potentially negative FGF23 effects upon cardiac structure and function, research needs to focus on and clarify relevant unsolved issues. Just to name a few, the community needs to prove how cardiac disease induces (rather than follows) FGF-23 secretion, to what degree cardiomyocytes may themselves produce FGF-23 in health and disease, whether such locally produced FGF-23 has a physiological role in (acute) myocardial damage; and whether or not (systemic) FGF23 excess itself directly drives the development of myocardial damage. Only when these questions have been answered, can we try to discuss whether and how to intervene on serum FGF-23 level.”

The authors apologize for this error and state that this does not change the scientific conclusions of the article in any way.

The original article has been updated.

## Conflict of interest statement

The authors declare that the research was conducted in the absence of any commercial or financial relationships that could be construed as a potential conflict of interest.

